# Advances in Group-10 Transition Metal Dichalcogenide PdSe_2_-Based Photodetectors: Outlook and Perspectives

**DOI:** 10.3390/s24186127

**Published:** 2024-09-22

**Authors:** Tawsif Ibne Alam, Kunxuan Liu, Sumaiya Umme Hani, Safayet Ahmed, Yuen Hong Tsang

**Affiliations:** 1 Shenzhen Research Institute, The Hong Kong Polytechnic University, Shenzhen 518057, China; tawsif-ibne.alam@connect.polyu.hk (T.I.A.); kunxu.liu@polyu.edu.hk (K.L.); sumaiya.hani@connect.polyu.hk (S.U.H.); 2 Department of Applied Physics, Materials Research Center, Photonics Research Institute and Research Institute for Advanced Manufacturing, The Hong Kong Polytechnic University, Hung Hom, Kowloon, Hong Kong, China; 3 Department of Physics, Oregon State University, Corvallis, OR 97331, USA; safayet.ahmed@oregonstate.edu

**Keywords:** PdSe_2_, palladium diselenide, TMDC photodetector, photothermoelectric, ambipolar, THz, ultrafast, ultrabroadband, photovoltaic

## Abstract

The recent advancements in low-dimensional material-based photodetectors have provided valuable insights into the fundamental properties of these materials, the design of their device architectures, and the strategic engineering approaches that have facilitated their remarkable progress. This review work consolidates and provides a comprehensive review of the recent progress in group-10 two-dimensional (2D) palladium diselenide (PdSe_2_)-based photodetectors. This work first offers a general overview of the various types of PdSe_2_ photodetectors, including their operating mechanisms and key performance metrics. A detailed examination is then conducted on the physical properties of 2D PdSe_2_ material and how these metrics, such as structural characteristics, optical anisotropy, carrier mobility, and bandgap, influence photodetector device performance and potential avenues for enhancement. Furthermore, the study delves into the current methods for synthesizing PdSe_2_ material and constructing the corresponding photodetector devices. The documented device performances and application prospects are thoroughly discussed. Finally, this review speculates on the existing trends and future research opportunities in the field of 2D PdSe_2_ photodetectors. Potential directions for continued advancement of these optoelectronic devices are proposed and forecasted.

## 1. Introduction

**Figure 1:**
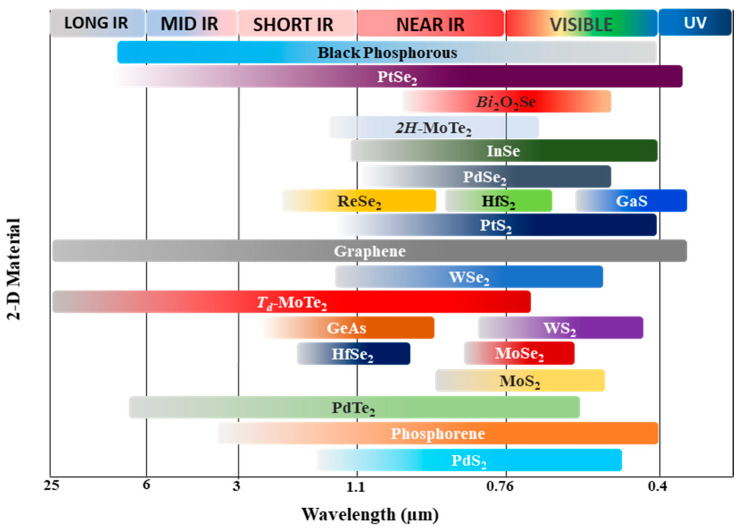
Spectral Response of some 2-D materials used in photodetection. Spectral response range data have been adopted from [23,25,26,27,28,29,30].

The advent of mechanically cleaved graphene from graphite has sparked a surge of research into layered low-dimensional materials [1]. Since then, a wide array of 2-dimensional layered materials (2DLMs) has been extensively studied, exhibiting a diverse range of captivating physical and chemical properties—semiconducting [2,3], semi-metallic, insulating [4,5], superconducting [6,7], and magnetic [8,9], to name a few. Of particular interest to researchers is the semiconducting nature of 2DLMs, as they can be tuned by adjusting the number of layers. This makes them a promising candidate for pioneering atomically thin electronics and optoelectronics. Moreover, the recent advancements in conventional bottom-up synthesis and patterning of large-area van der Waals (vdW) 2DLMs [10] have demonstrated their potential to be utilized as standalone semiconductors, as well as integrated with existing silicon technology [11,12,13,14]. Among the most widely studied semiconductor materials are the transition-metal dichalcogenides (TMDCs)—a family of compounds with the generic formula MX_2_, where M is a transition metal element and X is a chalcogen. The air-stable semiconducting TMDCs from group 6 of the periodic table, such as MoS_2_, WSe_2_, MoSe_2_, and WS_2_, have been extensively explored [15], as a single sheet of MoS_2_ has been predicted to absorb 5–10% of incident illumination in the visible wavelength [16]. These materials exhibit a fascinating layer-dependent behavior, where the bandgap increases and transitions from indirect to direct as the 2D layer approaches the monolayer limit [15,17]. This property is particularly important for optoelectronics, as the bandgap of the absorber layer directly determines the spectral sensitivity of the optoelectronic device. As illustrated in Figure 1, the vast library of 2DLMs offers a wide range of spectral response possibilities, simply by selecting the appropriate absorber material. More recently, group-10 TMDCs (M = Pd, Pt; X = Te, Se, S) have garnered significant attention in electronics and optoelectronics due to their promising traits, including broadband absorption, high carrier mobility [18], ambipolar transport properties [19,20], and long-term air stability [21]. PdSe_2_ stands out among the group-10 TMDCs due to its unique layer-dependent properties. As the number of layers is tuned, the material’s bandgap evolves from narrow to moderate, while maintaining appreciable ambipolar transport characteristics. This makes PdSe_2_ an ideal candidate for highly sensitive photodetectors and electronics. Furthermore, PdSe_2_ exhibits in-plane optical anisotropy, providing an additional degree of freedom that can be leveraged in devices with nonlinear optical phenomena and polarized photo-sensing applications. Fascinatingly, PdSe_2_ also exhibits optical response beyond its bandgap range, a behavior linked to its thermal properties [22]. This opens up the possibility of utilizing PdSe_2_ in miniaturized thermal sensing applications, further expanding its potential for optoelectronic and sensing technologies. PdSe_2_-based optoelectronics, particularly photodetectors, have the potential to be a game-changing technology for low-dimensional material-based detector applications. Other competing technologies for optoelectronics from group-10 TMDCs include PtSe_2_, PtTe_2_, PdTe_2_, and PdS_2_. Among them, PtSe_2_ and its heterojunctions have been rigorously investigated showing broadband response (MIR photodetection), ultrafast response speed (in µs), with high responsivity, detectivity, and ambipolar carrier mobility comparable to PdSe_2_-based optoelectronic devices and their heterostructures [23,24]. However, to provide a comprehensive understanding of PdSe_2_ and their heterostructures for photodetection mechanism, we only focus on PdSe_2_-based photodetectors in this article. This review aims to highlight the key material features, properties, synthesis, and characterization of PdSe_2_ devices relevant to photodetection. Additionally, it provides a perspective on future research directions toward PdSe_2_-based photosensors for real-life applications.

## 2. Figure of Merits and Types of PdSe_2_ Photodetectors

### 2.1. Figure of Merits (FoM) of PdSe_2_ Photodetectors

Photodetector performance is commonly evaluated using several key figures of merit (FoMs). These standardized FoMs allow for the evaluation and comparison of one photodetector to another, as well as assessing their suitability for specific applications. Table 1 outlines the most widely used FoMs for 2D-layered-material (2DLM)-based photodetectors, which are useful for assessing PdSe_2_-based photosensors. It provides a brief description, the mathematical expression, and the reporting units for each FoM.

### 2.2. Types of PdSe_2_ Photodetectors

**Figure 2:**
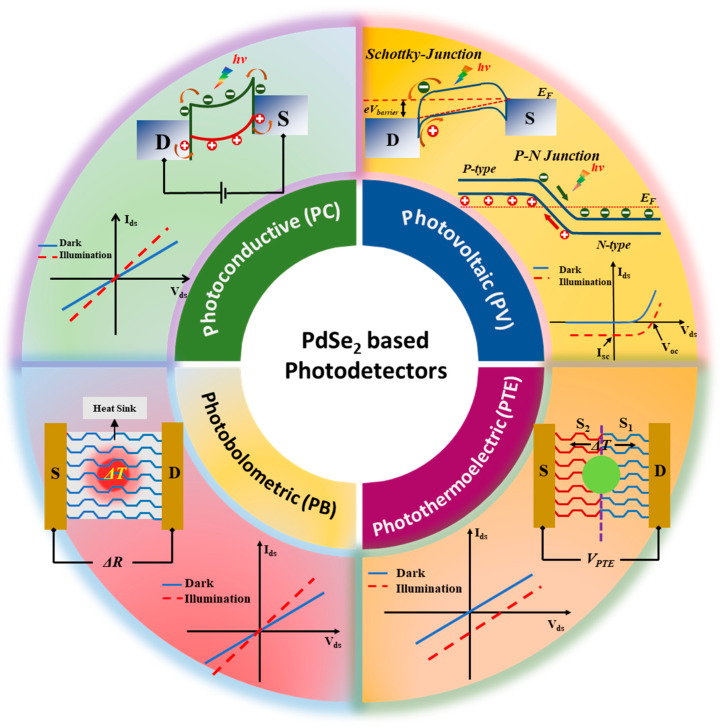
Broad classification of PdSe_2_-based photodetectors and their outlying internal mechanisms.

PdSe_2_ photodetectors can be broadly categorized into four types based on the literature: photovoltaic, photoconductive, photothermoelectric, and photobolometric, as illustrated in Figure 2. The specific photodetection mechanism in a low-dimensional material-based detector can be a standalone phenomenon or a combination of these effects, depending on the internal potential developed or external applied potential, the internal light-matter interaction mechanism within the 2D layered material, and the distribution of the input light flux density.

#### 2.2.1. Photovoltaic Type Photodetectors

In a photovoltaic-type photodetector, the built-in electric field that enables the photovoltaic effect (PVE), originates from a p-n junction [31] or a Schottky junction [32] at the semiconductor-metal interface. This internal field separates the photogenerated electron-hole (e-h) pairs.

Strategies to develop the necessary built-in electric field in 2DLM-based detectors include the following:

- Creating vertical or lateral heterojunctions [33,34];
- Chemical doping [35,36];
- Carefully selecting metals with different work functions [37,38].

**Figure 3:**
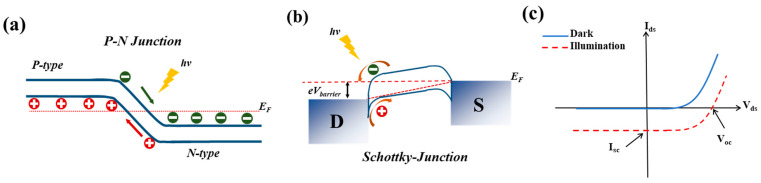
Energy band alignment in a typical (**a**) p-n junction (**b**) Schottky junction. (**c**) Typical *I_d_-V_d_* characteristics of a photovoltaic-type photodetector.

When the 2DLM active channel is illuminated with photons exceeding the bandgap energy, the absorbed photons excite e-h pairs that are then separated by the internal electric field due to the band bending at the p-n or Schottky junction (Figure 3a,b). This generates a short-circuit current (*I_sc_*) in the device (Figure 3c). The photocurrent direction is determined by the built-in potential. If the circuit is left open, the separated e-h pairs will instead create an open-circuit voltage (*V_oc_*) (Figure 3c). These photovoltaic photodetectors typically exhibit rectifying *I_d_-V_d_* characteristics and can operate without any bias or under reverse bias, enabling low dark currents and high quantum efficiencies.

#### 2.2.2. Photoconductive and Photo-Gated Photodetectors

**Figure 4:**
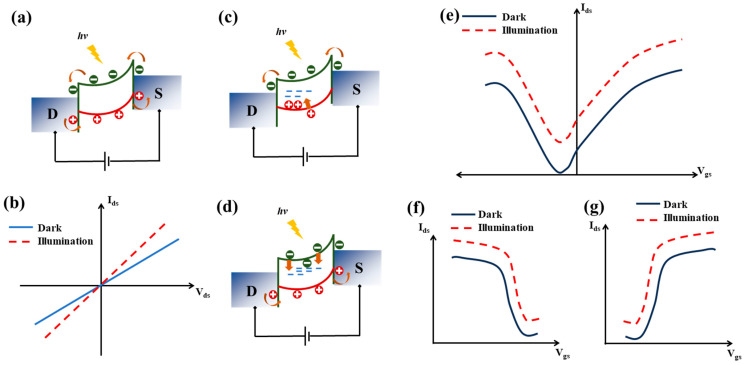
(**a**) Typical photoconductive effect in a 2DLM-based photodetector. The band bending and charge carrier extraction process requires an applied external bias voltage. (**b**) *I_d_-V_d_* characteristics of a typical photoconductive photodetector. (**c**) Typical photogated photodetector showing n-type characteristics due to p-type charge trapping. (**d**) Typical photogated photodetector showing p-type characteristics due to n-type charge trapping. (**e**) Transfer characteristics of a typical ambipolar photoconductive photodetector. (**f**) Transfer characteristics of a typical n-type carrier dominant photodetector. (**g**) Transfer characteristics of a typical p-type carrier dominant photodetector.

Photodetectors exhibiting the photoconductive effect (PCE) show an increase in free charge carriers when illuminated, typically in a semiconductor channel or photoconductor (PC) material. This rise in free carrier concentration lowers the channel’s resistance. Under an applied bias voltage (*V_ds_*), these excess charge carriers are swept apart, generating a photocurrent (*I_photo_*) (Figure 4a). The photocurrent is defined as the difference between the current under illumination (*I_light_*) and the dark current (*I_dark_*). When the channel material is illuminated with photons exceeding its bandgap energy, the absorbed photons create electron–hole (e-h) pairs. These carriers are then driven by *V_ds_*, causing the current to exceed the dark current due to the increased charge carrier concentration. Unlike photovoltaic-type detectors, the photocurrent in photoconductive device requires an applied bias voltage to be generated. As a result, these detectors do not exhibit a *V_oc_* or *I_sc_*, as illustrated in their typical *I_d_-V_d_* characteristics (Figure 4b).

Photo-gated photodetectors are a class of photoconductive devices, often considered phototransistors, which utilize the photogating effect. Under illumination, the typical photoconductive generation of electron–hole pairs occurs. However, the application of a gate bias causes charge-trapping states to function as a localized floating gate, significantly modulating the channel conductance [39,40]. When photogenerated holes are trapped in positively charged hole-trapping sites, the remaining free electrons can roam for a longer time before recombining. This provides high gain and n-type doping characteristics (Figure 4c). The opposite occurs when electrons are trapped, resulting in p-type behavior (Figure 4d). This photogating mechanism allows for tuning the channel conductivity. By carefully designing the device, such as tuning the metal work function, photo-gated detectors can exhibit ambipolar transport characteristics (Figure 4e) or be optimized for unipolar operation [41,42] (Figure 4f,g). However, the charge trapping and detrapping processes leads to a sluggish photoresponse, which is a trade-off for enhanced photoresponsivity and gain [43,44].

#### 2.2.3. Photothermoelectric (PTE) Photodetectors

**Figure 5:**
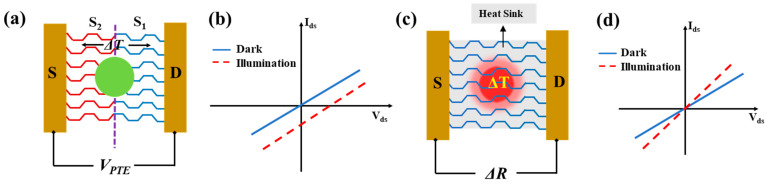
(**a**) Typical PdSe_2_-based PTE photodetector. Localized light spot (green color) generates a temperature gradient across the entire PdSe_2_ channel material, from the drain to source electrodes, developing a photothermoelectric potential (*V_PTE_*). (**b**) *I_d_-V_d_* characteristics of a photo thermoelectric type photodetector. (**c**) Typical PdSe_2_-based PB photodetector. Uniform light illumination results in a temperature-induced resistance change (Δ*R*) of the channel material. (**d**) Typical *I_d_-V_d_* characteristics of a PB-type detector.

Localized light irradiation of a 2DLM can create a thermal effect known as the photothermoelectric (PTE) effect. The localized illumination generates a temperature gradient (*ΔT*) across the semiconductor channel, as the spot size is smaller than the channel dimensions [37]. This temperature differential (*ΔT*) induces a photothermoelectric potential difference (*V_PTE_*) across the channel ends, based on the difference in Seebeck coefficients (*S_1_* and *S_2_*) of the material: *V_PTE_* = (*S_2_ − S_1_*)·*ΔT*. The units of *V_PTE_* are V/K. The development of this photo-induced thermoelectric potential, as shown in the typical *I_d_-V_d_* characteristics in Figure 5b, allows PTE-based photodetectors to operate in a self-powered mode, without requiring any external bias voltage. The electrical conductivity of the channel material is closely related to the Seebeck coefficients *S_1_* and *S_2_* through the Mott formula: S = $\frac{\pi^{2}{k_{B}}^{2}T_{e}}{3q}\frac{1}{\sigma }\frac{\partial \sigma }{\partial \varepsilon_{F}}$ where *T_e_* is electron temperature, and the derivative of the conductivity σ with respect to energy *ε_F_* must be evaluated at fermi energy [45,46]. It is important to note that global illumination can also cause the PTE effect in 2DLM, provided there is a strong absorption gradient within the channel to generate the necessary temperature differential and form *V_PTE_*, typically in the range of a few microvolts to millivolts. The sign of the photogenerated current depends on the difference in Seebeck coefficients of the channel material localities and the carrier polarity in the channel.

#### 2.2.4. Photobolometric Photodetectors

Thermal effect-induced carriers can also be generated by uniform light-induced heating of a channel material due to the Photobolometric Effect (PBE). During the PBE effect, the heat-sensitive channel material will undergo a change in its resistivity. The thermal resistance (*R_tr_*) is associated with the rate of change in the temperature with respect to the incident power (*R_tr_* = *dT*/*dP*). Bolometric photodetectors are not self-powered like photo thermoelectric detectors and hence require an external bias voltage. The external bias voltage linearly scales the heat-induced photocurrent generated by a bolometric photodetector (Figure 5d).

## 3. Properties of Group-10 TMDC PdSe_2_

### 3.1. Structural Characteristics

**Figure 6:**
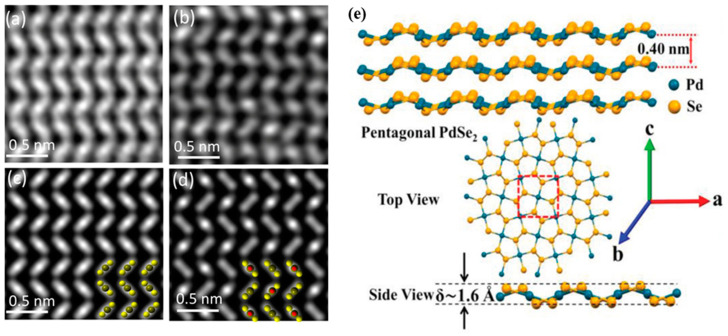
(**a**–**d**) Z-contrast STEM pictures of few-layer PdSe_2_ crystals (**top row**) and comparable simulated images of PdSe_2_ (**bottom row**) illustrate the atomic resolution structure of the material. There are even (**a**,**c**) and odd (**b**,**d**) numbers of layers, respectively. Atomic models of the relevant STEM pictures are shown in the insets in (**c**,**d**). Reprinted with permission from ref. [21] © 2017 American Chemical Society (**e**) crystal structure of 2D PdSe_2_ monolayer and few-layer systems. Reprinted with permission from ref. [49] © 2018 WILEY-VCH Verlag GmbH & Co. KGaA, Weinheim, Germany.

The most widely studied PdSe_2_ phase is in the 2DLM form that exhibits pentagonal rings with a puckered structure, analogous to black phosphorus (BP). This unique puckered configuration is responsible for the material’s exotic, polarization-dependent anisotropic optoelectronic characteristics. While other reported polymorphs of PdSe_2_ [18,47], have also been investigated, this discussion will focus specifically on the phase possessing the puckered pentagonal ring structure in the 2DLM form. In this puckered phase, each palladium (Pd) atom is bonded to four selenium (Se) atoms within the same atomic layer, while neighboring Se-Se bonds are formed through covalent interactions. The bulk crystalline structure of this PdSe_2_ polymorph displays Pbca symmetry [48], belonging to the *D_2h_* point group family, with an orthorhombic lattice. The unit cell of this PdSe_2_ phase comprises four Pd and eight Se atoms, with lattice parameters of a = 5.7 Å, b = 5.87 Å, and c = 7.69 Å, as reported in [21]. The individual layers of PdSe_2_ are held together along the c-axis via van der Waals interactions, with a van der Waals gap spacing of 0.40 nm between adjacent layers. The puckered thickness of a single PdSe_2_ layer is approximately 1.6 Å. Figure 6a–d provides a visual illustration of the z-contrast HRTEM image of a few layers of PdSe_2_ and its simulated counterparts while the vdW phase PdSe_2_ along with its crystal structure is illustrated in Figure 6e.

### 3.2. Raman Spectroscopic Characterization

**Figure 7:**
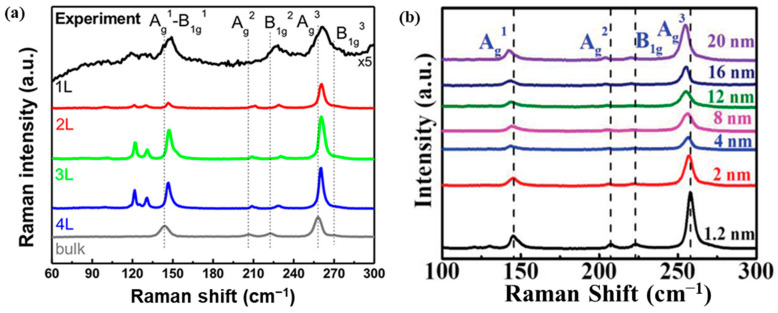
(**a**) Exfoliated PdSe_2_ nanosheets’ layer-dependent Raman spectra (monolayer to bulk) at 532 nm excitation laser wavelength. Reprinted with permission from ref. [21] © 2017 American Chemical Society (**b**) Raman spectra of PdSe_2_ films synthesized via CVD with varying thickness. Reprinted with permission from ref. [49] © 2018 WILEY-VCH Verlag GmbH & Co. KGaA, Weinheim.

Raman spectroscopy studies have revealed strong interlayer interactions within PdSe_2_ crystals, with distinct Raman peak shifts observed under variable layer thickness [21]. Raman analysis of exfoliated PdSe_2_ nanosheets, typically consisting of around 5 layers, have been reported with the presence of six Raman peaks. These peaks can be attributed to three ***A*** modes (***A_g_^1^***, ***A_g_^2^***, ***A_g_^3^***) and three ***B_1g_*** modes (***B_1g_^1^***, ***B_1g_^2^***, ***B_1g_^3^***), corresponding to in-plane and out-of-plane vibrational modes, respectively [21,50] illustrated in Figure 7a. In contrast, Raman measurements on wafer-scale PdSe_2_ sheets have been reported with the observation of only four distinct Raman modes, located at approximately 144 cm^−1^, 205 cm^−1^, 222 cm^−1^, and 255 cm^−1^ (Figure 7b). These modes can be assigned to ***A_g_^1^*** (with a contribution from ***B_1g_^1^***), ***A_g_^2^***, ***B^1^_g_***, and ***Ag^3^*** vibrations, respectively [49,51]. The slight discrepancy between the Raman spectra of exfoliated and wafer-scale PdSe_2_ samples can be attributed to the merging of certain Raman modes, such as ***A_g_^1^*** with ***B_1g_^1^***, and ***A_g_^3^*** with ***B_1g_^3^***, in the wafer-scale samples [50,52]. These observations highlight the sensitivity of the PdSe_2_ Raman modes to the material’s layer thickness and overall structural characteristics, based on the process in which the 2DLM is synthesized.

### 3.3. Anisotropic Response to Optical Stimulus

**Figure 8:**
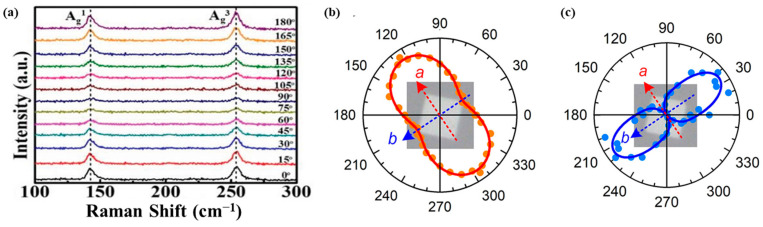
(**a**) Angle-resolved polarized Raman spectra of 2D PdSe_2_. Reprinted with permission from ref. [54]. © 2019 The Authors. Published by WILEY-VCH Verlag GmbH & Co. KGaA, Weinheim. (**b**,**c**) Polar plots for the 4L PdSe_2_ assessed under the copolarization configuration as a function of the azimuthal angle φ, showing Raman intensity of the A_g_^1^ mode intensity (**b**) and second harmonic intensity (**c**) (a [red arrow] and b [blue arrow] are crystallographic axis of the flake). Reprinted with permission from ref. [53] © 2020 American Chemical Society.

The distinct pentagonal structure of PdSe_2_ crystal is projected to impart pronounced in-plane anisotropy in their electrical, mechanical, and optical properties. Angle-resolved polarized Raman spectroscopy has emerged as a powerful technique to probe and characterize the optical anisotropy of these two-dimensional materials. Polarized Raman studies on PdSe_2_ have revealed a notable angular dependence of the Raman-active modes. Specifically, the intensities of the ***A_g_^1^*** and ***A_g_^3^*** modes are observed to gradually decrease from 0° to 90° with respect to the polarization angle, followed by a subsequent increase from 90° to 180°. This angular modulation of the ***A_g_^1^*** and ***A_g_^3^*** peak intensities provides direct evidence of the structural anisotropy inherent to the PdSe_2_ crystal lattice obtained by CVD [53,54] (Figure 8a,b). This anisotropy can be attributed to the unique pentagonal coordination of the atoms within the layered PdSe_2_ structure, with potential implications for the material’s electrical, mechanical, and optoelectronic performance.

Complementing these polarized Raman studies, polarization-resolved second harmonic generation (SHG) measurements have provided additional insights into the crystallographic anisotropy of PdSe_2_. It is well established that the even-numbered layer PdSe_2_ belongs to the *C_2v_* point group symmetry, while odd-numbered layers correspond to the *C_2h_* symmetry [55]. As a result, even-layered PdSe_2_ crystals exhibit a second-order nonlinear optical response, which can be probed through polarization-dependent SHG experiments. More precisely, for even-layered PdSe_2_, the co-polarized SHG response (the second-order nonlinear susceptibility *χ*^(*2*)^ of even-layered PdSe_2_) is found to be maximized when the incident electric field is aligned with the b-axis of the crystal [53] (Figure 8c). This direct correlation between the SHG response and the crystallographic orientation provides additional evidence of the strong optical anisotropy in these layered PdSe_2_ materials. Together, the angle-resolved polarized Raman and SHG studies offer complementary insights into the anisotropic optical properties of PdSe_2_, stemming from its unique pentagonal layered structure. These techniques pave the way for a comprehensive understanding of the “structure to characteristic” relationships in this promising 2DLM.

### 3.4. Layer Dependent Optical Absorption and Bandgap Evolution

**Figure 9:**
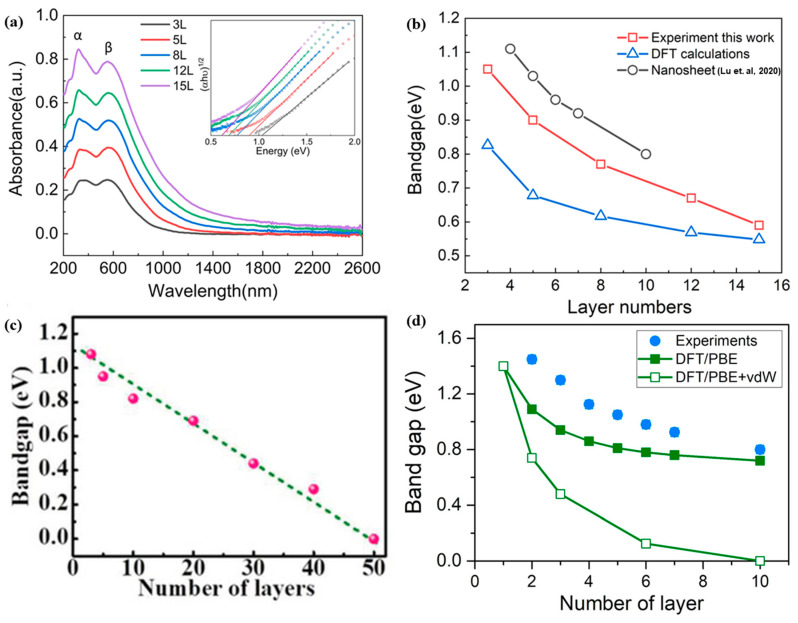
(**a**) absorption spectra and (**b**) Bandgap evolution of PdSe_2_ films with layer numbers ranging from 3L to 15L. The two major absorption peaks of PdSe_2_ films are represented by α and β, with corresponding tauc plots displayed in the inset. Experimental data of nanosheets was adopted from [53]. Adapted with permission from ref [56]. Creative Commons Attribution CC BY license. (**c**) The optical bandgaps extracted from Tauc plots for PdSe_2_ with different layer numbers. Reprinted with permission from ref. [49] © 2018 WILEY-VCH Verlag GmbH & Co. KGaA, Weinheim. (**d**) Optical band gap as a function of layer number determined by Tauc plot, along with representations of DFT-based assumptions. Reprinted with permission from ref. [53] © 2020 American Chemical Society.

The unique layered structure of PdSe_2_ confers not only pronounced in-plane anisotropy in their optical properties, as evidenced by polarized Raman and SHG studies, but also a remarkable thickness-dependent evolution of the optical bandgap. Optical absorption measurements on large-area, centimeter-scale PdSe_2_ films with thicknesses ranging from 3 to 15 layers have revealed two distinct excitonic peaks, labeled α and β, in the absorption spectra (Figure 9a). Notably, the corresponding optical bandgap, as determined by Tauc plot extrapolations, exhibits a systematic decrease with increasing layer number (Figure 9b) [56]. This experimentally observed bandgap reduction, is also consistent from separate investigations with bandgap evolution from 1.08 eV for 3-layer PdSe_2_ to 0.29 eV for 40-layer PdSe_2_ (Figure 9c), in line with density functional theory (DFT) calculations and highlights the semiconducting nature of these layered materials. Interestingly, as the layer number reaches 50, the bandgap closes completely, transitioning the 2DLM to a quasi-metallic phase [49]. More in-depth electronic and optoelectronic investigations in recent reports reveal that the bandgap may exist for bulk PdSe_2_ within the vicinity of 0.3 eV–0.5 eV [57,58,59]. This dramatic evolution of the electronic structure with thickness underscores the unique dimensionality-dependent properties of PdSe_2_ thin films. The thickness-dependent bandgap tunability has also been observed in chemical vapor deposition (CVD)-grown PdSe_2_ flakes, using a microscope-based transmission spectroscopy approach [53]. In this case, the bandgap was found to vary from 1.43 eV for 2-layer flakes to 0.8 eV for 10-layer flakes (Figure 9d), in good agreement with DFT estimates. Notably, the observed bandgaps in these CVD-grown PdSe_2_ flakes are indirect in nature, providing further insights into the electronic structure of this 2DLM. The ability to continuously tune the optical bandgap of PdSe_2_ through thickness control presents exciting opportunities for the development of atomically thin, layer-engineered optoelectronic devices. The combination of anisotropic optical properties and bandgap tunability in PdSe_2_ underscores its promise as a versatile channel material for exploring the interplay between dimensionality, structure, and electronic behavior in the 2D limit.

### 3.5. Carrier Transport Characteristics and Carrier Mobility

**Figure 10:**
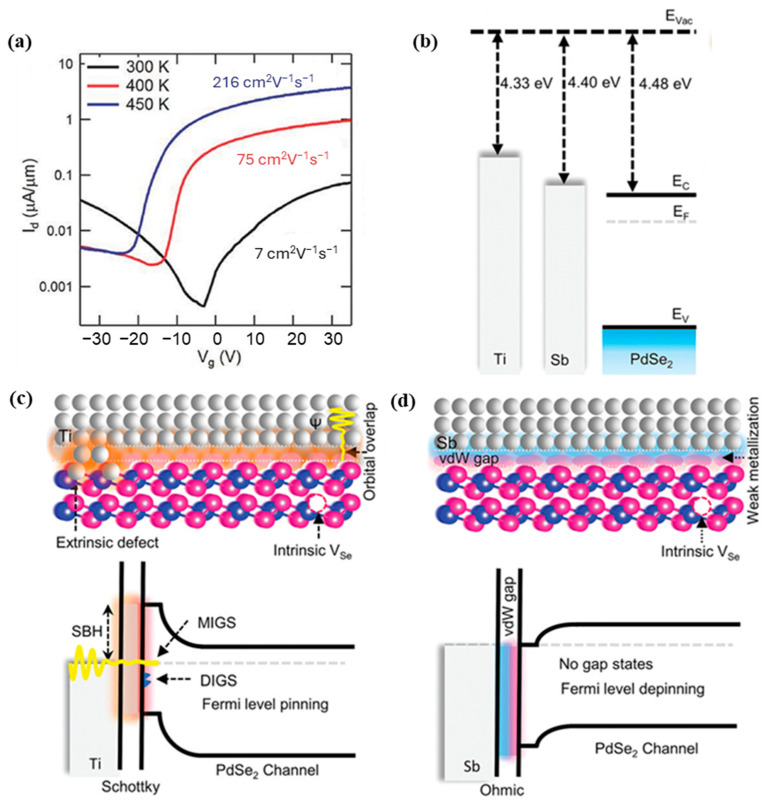
(**a**) *I_d_-V_g_* characteristics demonstrating the gradual transition following annealing at 400 K (red curve) and 450 K (blue curve) from an ambipolar transport (black curve) to an electron–transport dominated system. Adapted with permission from ref. [61]. © 2017 WILEY-VCH Verlag GmbH & Co. KGaA, Weinheim. Ti–PdSe_2_ and Sb–PdSe_2_ contacted FETs (**b**) PdSe_2_ few-layer pre-contact energy band diagrams with Ti and Sb. (**c**) Ti–PdSe_2_ and (**d**) Sb–PdSe_2_ contacts’ schematic cross-sectional views of the interface interaction with band diagrams post-contact. Adapted with permission from ref. [19] © 2023 Wiley-VCH GmbH.

As discussed earlier, PdSe_2_ has demonstrated structural anisotropy, suggesting the potential for anisotropic electronic behavior. The 2DLMs with high charge carrier mobility are favorably desirable for photodetector applications, as this enables more efficient collection of charge carriers and improved device speed. Existing reports on the transport characteristics of PdSe_2_ have revealed ambipolar conduction, whereby the material can exhibit both n-type and p-type behavior, which can be tuned through vacuum annealing or annealing in an inert environment [60]. Multiple PdSe_2_ field-effect transistors (FETs) fabricated with gold (Au) contacts, without any annealing treatment, have exhibited maximum (average) electron field-effect mobilities (*µ_e_*) of 54 cm^2^V^−1^s^−1^ (17 cm^2^V^−1^s^−1^) and hole mobilities (*μ_h_*) of 14 cm^2^V^−1^s^−1^ (7 cm^2^V^−1^s^−1^) [19]. These mobility values are notable in comparison to the diverse range of mobility (1–50 cm^2^V^−1^s^−1^) reported for the widely studied MoS_2_ material. Vacuum annealing of the PdSe_2_ FETs at 450 K resulted in an increase in electron mobility, reaching 216 cm^2^V^−1^s^−1^ [61], and a transition towards n-type dominant behavior (Figure 10a). Further improvements in room-temperature electron mobility, up to 383 cm^2^V^−1^s^−1^, have been reported for PdSe_2_ devices with van der Waals (vdW) contacted antimony (Sb) source-drain electrodes [19]. The vdW gap between the Sb contacts and the PdSe_2_ channel led to Fermi level depinning and effective work function alignment, minimizing the Schottky barrier height (Figure 10b–d) and reducing the contact resistance to as low as 0.55 kΩ.

## 4. PdSe_2_ Photodetectors: Fabrication Methods and Their Performance

2D PdSe_2_ can be utilized as the channel material for high-performance photodetectors, which can be fabricated using a variety of synthesis techniques. The most widely reported methods for the preparation of high-quality 2D PdSe_2_ include chemical vapor deposition (CVD) and mechanical exfoliation of crystals obtained from chemical vapor transport (CVT) [62] or self-flux growth processes [63]. The CVD technique has enabled large-area growth of continuous PdSe_2_ films as well as discrete flakes, which have been subsequently employed in the fabrication of pristine and heterostructure-based PdSe_2_ photodetectors. Heterostructure photodetectors, wherein the PdSe_2_ channel is sandwiched between other 2D materials, offer a means of enhancing the intrinsic capabilities of the PdSe_2_ layer. Such heterostructure configurations have been shown to provide benefits such as lowered dark and suppressed noise currents [64], broadened spectral response [22], improved photogenerated charge separation [31], and enhanced switching speeds [38], among other performance enhancements.

In the following sections, a categorical exploration of the various PdSe_2_-based photodetector structures will be presented, classified based on the employed fabrication methodologies with a discussion on how their performance is affected.

### 4.1. CVD Grown PdSe_2_ for Photodetectors

#### 4.1.1. Standalone CVD Grown PdSe_2_ Photodetectors

**Figure 11:**
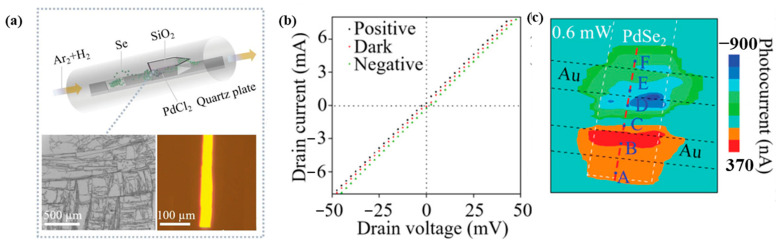
(**a**) Schematic illustration of the CVD configuration for synthesizing PdSe_2_ nanosheets on Si/SiO_2_ with the precursors of PdCl_2_ and Se. (**b**) *I_d_-V_d_* characteristics of the device [dark (red solid line) and illumination (black and cyan solid line]. (**c**) SPCM images of the PdSe_2_ photodetector at zero bias with 532 nm excitation. Adapted with permission from ref. [20]. © 2021 Wiley-VCH GmbH.

Photothermoelectric (PTE)-based PdSe_2_ was investigated by Li et al. [20], which was grown via chemical vapor deposition (CVD). Rather than relying on electron–hole separation, the observed distinctive photoresponse was attributed to the generation of an electron temperature gradient. The presence of a nonlocal photoresponse with zero bias provided direct evidence of the PTE effect. The synthesis method employed by the researchers involved placing a mixture of PdCl_2_ and NaCl powders in a crucible, with the SiO₂ substrate positioned face-down atop the crucible at a temperature of 750 °C. Separately, selenium powder was used as the source material and placed outside the hot zone at a temperature of 350 °C (Figure 11a). The fabricated devices exhibited ohmic contacts (Figure 11b) and scanning photocurrent microscopy (SPCM) mapping revealed that the photocurrent generation was confined solely to the contact regions (Figure 11c). This observation ruled out the involvement of photovoltaic and photo-Dember effects, suggesting that the PTE effect was solely responsible for the observed broadband photoresponse, which was not limited by the material’s bandgap. The PTE device recorded an astounding ultrafast response of 4 µs, making it one of the fastest-recorded PdSe_2_-based photodetectors. Zhang et al. [65] fabricated PdSe_2_-based bolometric photodetectors using low-temperature (200 °C) plasma-enhanced selenization of Pd films. The devices recorded a broadband response (365 nm–2200 nm) with a response time of < 1.2s.

#### 4.1.2. CVD-PdSe_2_ Hybrid Heterostructure-Based Photodetectors

**Figure 12:**
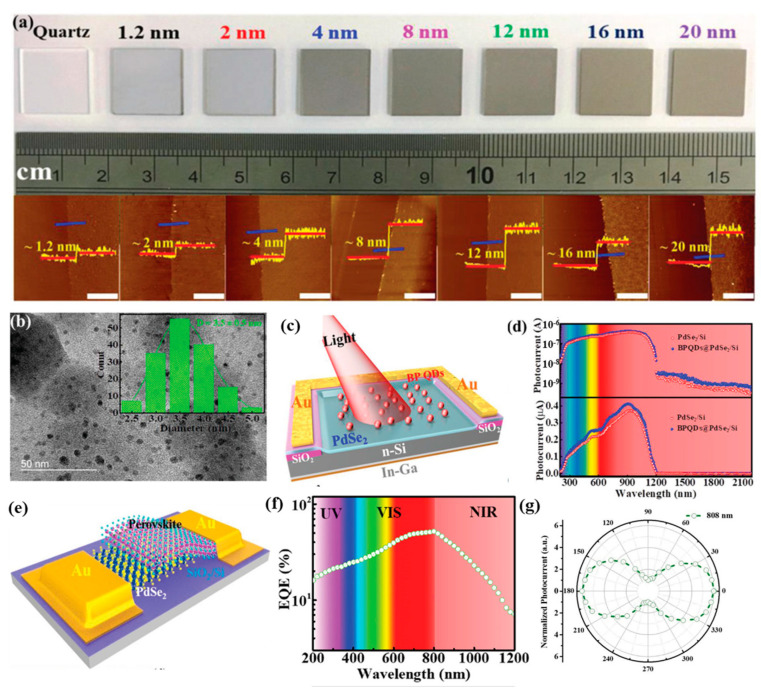
(**a**) Digital camera photo of PdSe_2_ films with different thicknesses grown on the quartz; the bottom pictures show the corresponding AFM images. The blue line represents the direction of height measurement and red line denotes the height profile (**b**) TEM image of BPQDs with an average diameter of 3.5 ± 0.5 nm. Statistical analysis of the lateral sizes of 160 BPQDs (inset) (**c**) Schematic illustration of a BP-QD-PdSe_2_/Si detector, (**d**) The spectral response of PdSe_2_/Si with and without BPQDs decoration. Adapted with permission from ref. [49] © 2018 WILEY-VCH Verlag GmbH & Co. KGaA, Weinheim. (**e**) Schematic illustration of PdSe_2_/perovskite hybrid junction detector. (**f**) The wavelength-dependent external quantum efficiency (EQE) of PdSe_2_/perovskite device at zero bias. (**g**) The evolution of photocurrent as a function of different polarized angle. Reprinted with permission from ref. [54] © 2019 The Authors. Published by WILEY-VCH Verlag GmbH & Co. KGaA, Weinheim.

Large-area, centimeter-scale PdSe_2_-based hybrid photodetectors can be fabricated using a two-step selenization method [49,51]. In the first step, palladium (Pd) is deposited onto a substrate using magnetron sputtering. The as-deposited Pd film is then annealed in a tube furnace under a flow of vaporized selenium and argon gas. This process converts the Pd film into a polycrystalline PdSe_2_ layer, which can be grown to the desired thickness on a variety of substrate materials. A layer-dependent evolution of large area PdSe_2_ thin film on quartz substrate with corresponding atomic force microscopy (AFM) micrograph is illustrated in Figure 12a. These PdSe_2_ films can be utilized to fabricate vertical hybrid photodetector structures, where the PdSe_2_ layer is combined with other conventional materials or perovskites. One such example is the PdSe_2_-Silicon Nanowire Array (PdSe_2_-SiNWA) device, where the PdSe_2_ layer is transferred onto a silicon nanowire array structure. These PdSe_2_-SiNWA photodetectors have demonstrated impressive performance, with a high responsivity of 726 mA/W, a high specific detectivity of 3.19 × 10^14^ Jones, and an ultra-broadband spectral response ranging from 0.2 to 4.6 μm, along with a dichroic ratio (polarization sensitivity) of 75. Similar fabrication approaches using the two-step selenization method have also been employed to create other types of PdSe_2_-based heterojunction photodetectors, PdSe_2_-Perovskite (with broadband polarization sensitivity of ~6.04) [54], PdSe_2_-Si or Black Phosphorous Quantum Dot-PdSe_2_-Si (BPQD-PdSe_2_-Si) (for near-infrared to mid-infrared detection) [49] and PdSe_2_-GaN (for polarized UV light detection) [66]. Amongst them, the hybrid PdSe_2_-Perovskite heterojunction detector exhibited detection between 200 and 1550 nm with appreciable external quantum efficiency due to charge trapping in the perovskite layer, despite having photovoltaic characteristics, while the BPQD-PdSe_2_-Si hybrid junction exhibited self-powered detection with an ultra-broadband range of 200 nm–3044 nm with response speeds of less than 45 µs.

#### 4.1.3. CVD Grown PdSe_2_-2D Heterostructure-Based Photodetectors

**Figure 13:**
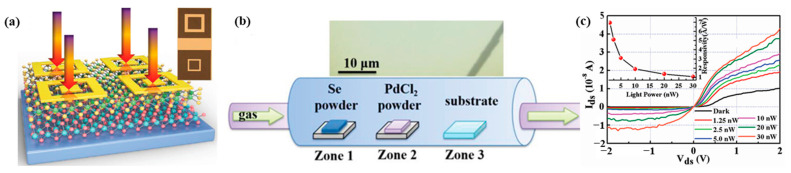
(**a**) The schematic image of the MoS_2_/PdSe_2_ photodetector, and the inset is an optical image of the device (**b**) The schematic diagram of the CVD system and the inset is the optical image of the grown uniform PdSe_2_ film. (**c**) I_ds_-V_ds_ characteristics of MoS_2_/PdSe_2_ photodetector at 830 nm with zero gate bias under variable incident light power. Reproduced with permission from ref. [67] © 2022 Wiley-VCH GmbH.

Reports have described the fabrication of PdSe_2_-based 2DLM heterostructures grown via chemical vapor deposition (CVD) methods. For instance, one study [67] details the growth of PdSe_2_ on a sapphire substrate using PdCl_2_ and Se precursors in a three-zone furnace system. Utilizing an Ar/H₂ carrier gas, the Se source at 250 °C (zone-1) and PdCl₂ source at 500 °C (zone-2) were vaporized, and PdSe_2_ was subsequently synthesized on the sapphire substrate at 600 °C (zone-3). Using a similar approach, MoS₂ was grown on a separate substrate and then transferred onto the PdSe_2_ film using a PMMA-based wet transfer technique to form large-area PdSe_2_-MoS_2_ heterostructures. Figure 13a shows a schematic illustration of the fabricated device while Figure 13b shows the setup of the 3-zone CVD synthesis system employed. Despite having a type II band alignment, the device did not show self-powered characteristics as illustrated in the *I_d_-V_d_* characteristics (Figure 13c).

**Figure 14:**
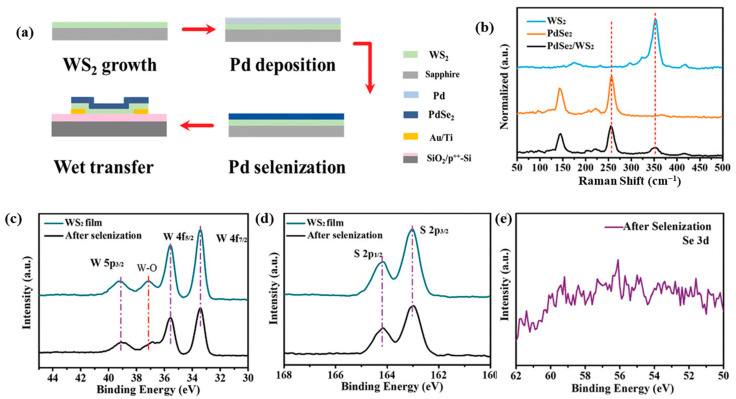
(**a**) Schematic of the device fabrication process. (**b**) Raman spectra of the stacked film extracted from the regions corresponding to WS_2_, PdSe_2_, and PdSe_2_/WS_2_ respectively. (**c**) XPS spectra of W 4f peak. (**d**) XPS spectra of S 2p peak. (**e**) XPS spectra of Se 3d peak. Adapted with permission from ref. [68]. © 2021 Wiley-VCH GmbH.

Conversely, a different method was employed to fabricate PdSe_2_-WS₂ heterostructures [68], which eliminated the need for wet transfer of the synthesized 2DLM. In this case, a WS₂ film was first grown on a sapphire substrate, followed by the deposition of Pd and subsequent selenization to complete the PdSe_2_-WS₂ heterostructure (Figure 14a). Raman spectroscopy and X-ray photoelectron spectroscopy (XPS) characterization before and after the selenization process confirmed the pristine quality of the WS₂ layer even after the formation of the PdSe_2_-WS_2_ heterostructure (Figure 14b–e). The device developed a type-I band alignment, and no photovoltaic effect was observed with only photoconductive effect dominating the device performance with an appreciable response time of 49 ms/90 ms at a bias of 2 V. On the contrary, Shi et al. [69] reported the fabrication of a PdSe_2_/MoS_2_ p-n heterojunction photodetector by transferring mechanically exfoliated MoS₂ onto CVD-grown PdSe_2_ films. This device exhibited a high responsivity of 2.7 A/W under zero-bias conditions, which was attributed to the built-in potential at the heterojunction. Additionally, the device demonstrated fast switching speeds of 193 μs (rise) and 96 μs (fall).

### 4.2. Mechanically Exfoliated PdSe_2_ Photodetectors

Mechanically exfoliated (M.E.) layers of PdSe_2_ have been extensively utilized in proof-of-concept photodetector devices. In this approach, a bulk PdSe_2_ crystal is loaded onto an adhesive tape and mechanically cleaved multiple times. The exfoliated layers are then transferred onto a SiO_2_/Si substrate by pressing the sticky tape onto the substrate and slowly removing it after a few minutes. A common subsequent step involves the use of electron beam lithography to define the source and drain regions, followed by the deposition of metallic contacts via techniques such as electron beam evaporation to complete the fabrication of pristine PdSe_2_-based photodetectors. An alternative, more mature method involves the deterministic transfer of PdSe_2_ layers onto pre-patterned drain/source electrodes on SiO_2_/Si substrates to form pristine photodetectors. Furthermore, 2D heterostructures can be created by deterministically transferring mechanically cleaved PdSe_2_ layers onto a previously exfoliated 2D material on a typical SiO_2_/Si substrate, using PDMS stamps. This deterministic transfer approach has been further refined and expanded to include the transfer of 2D materials from one substrate to another using polymers such as polymethyl methacrylate (PMMA), as well as thermoplastic materials like polyvinyl alcohol (PVA), polycarbonate (PC), and polypropylene carbonate (PPC).

#### 4.2.1. Standalone M.E. PdSe_2_ Photodetectors

One of the first reports of using PdSe_2_ as a sole channel material can be reported back to [70]. The device was constructed out of a PdSe_2_ flake connected with Ti/Au electrodes. The device responsivity could be modulated using the back gate to achieve very high photogains of 708 A/W at 1064 nm. The device, however, suffered from poor mobility and low linear dynamic range. Reports of THz detection with PdSe_2_ were also conveyed for a photovoltaic-type device using a hot carrier injection mechanism and asymmetric antenna type contact engineered photodetector [22]. The device portrayed a responsivity of 5 mA/W at 0.24 THz and a response speed of 7.5 µs with application in THz imaging.

**Figure 15:**
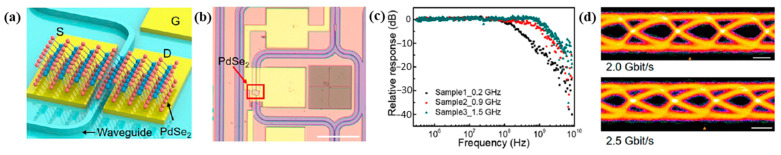
(**a**) Schematic (**b**) Optical micrograph illustration of waveguide integrated PdSe_2_ photodetector. (**c**) Measured frequency response of three PdSe_2_ photodetectors at 3 V. (**d**) Receiver eye diagram at a data rate of 2.0 and 2.5 Gbits^−1^ measured with PdSe_2_ photodetector. Reprinted with permission from ref. [71]. Copyright © 2022 American Chemical Society.

PdSe_2_ nanosheets exfoliated onto silicon-on-insulator (SOI) ridge waveguides were investigated for the development of integrated photodetectors operating in the telecommunications wavelength band (1260 nm to 1565 nm) [71]. The waveguide architecture is an attractive approach for designing photodetectors with both high responsivity and large bandwidth (Figure 15a,b). The fabricated devices exhibited excellent performance characteristics, with a high responsivity of 1758.7 mA/W and a 3 dB bandwidth of 1.5 GHz (Figure 15c). Furthermore, the devices demonstrated a data rate capability in the range of 2.5 Gbit/s, as evidenced by the clear eye-opening in the eye diagram (Figure 15d).

Zhong et al. [50] performed electronic and optoelectronic characterization of mechanically exfoliated 5-layer PdSe_2_ nanosheets in a field-effect transistor (FET) configuration. The researchers obtained a decent field-effect mobility of 1.8 cm^2^/Vs, indicating hole-dominant transport characteristics, and a fast response time of 11 ms (rise) and 6 ms (fall). Polarization-dependent photocurrent mapping of the PdSe_2_ nanosheet devices revealed a dichroic ratio of 1.9, demonstrating a polarization-dependent photoresponse. As the polarization angle was increased, the photocurrent and responsivity decreased, with a maximum responsivity of 3.5 mA/W.

#### 4.2.2. M.E. PdSe_2_-2D Heterostructure-Based Photodetectors

**Figure 16:**
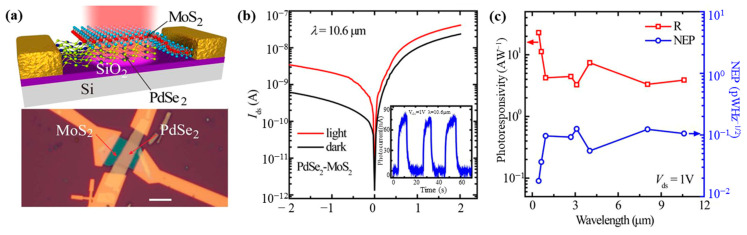
(**a**) Top panel: Schematic image, Bottom panel: optical photograph of o PdSe_2_−MoS_2_ infrared photodetector. (**b**) Log scale *I_d_−V_d_* characteristic under dark (black) and illuminated (red) conditions. Inset: Time-resolved photoresponse curves. (**c**) Biased (*V_d_* = 1 V) wavelength-dependent photoresponsivity R (red) and noise equivalent power (blue) of the photodetector under ambient air. Adapted with permission from ref. [72]. Copyright © 2019 American Chemical Society.

Long-wavelength infrared operation of PdSe_2_-MoS_2_-based heterostructure field-effect transistors (FETs) has been reported by [72] (Figure 16a). The response times were 74.5 ms (rise) and 91.3 ms (fall) in the 10.6 μm wavelength range (Figure 16b), and the devices showed a broad spectral response from 450 nm to 10.6 μm with exceptional performance, with regard to responsivity of 42.1 A/W (Figure 16c) and a detectivity of 1.10 × 10^9^ Jones at a wavelength of 10.6 μm. The authors noted that the use of Pd/Au contacts could facilitate a reduction in dark currents (Figure 16b), thereby improving the on/off ratio and overall device performance.

**Figure 17:**
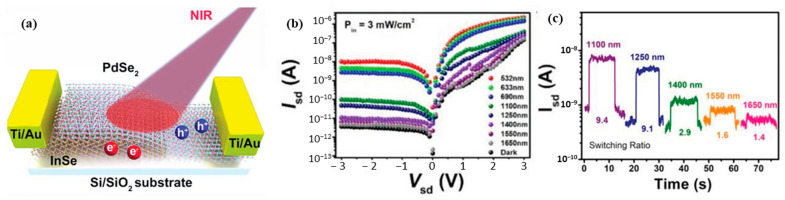
(**a**) Schematic illustration of InSe/PdSe_2_ vdWs photodetector. (**b**) log I-V characteristics of the device under various illumination wavelengths a fixed light power density of 3 mW/cm^2^. (**c**) Temporal response of the device under various illumination wavelengths with mentioned light on/off ratio. Reproduced with permission from ref. [73]. © 2021 Wiley-VCH GmbH.

Broadband photodetection has been reported for PdSe_2_-InSe heterostructures [73], spanning the visible to near-infrared (NIR) wavelength range (Figure 17a). These devices exhibited response speeds in the millisecond range and a high responsivity of 161 A/W in the NIR region (1250 nm). The device performance was further enhanced by gate-tunable photocurrent, indicating electron-dominated transport characteristics. Additionally, the PdSe_2_-InSe heterostructures demonstrated appreciable switching speeds and extended detection capabilities up to 1650 nm (Figure 17b,c), which exceeds the individual responses of the PdSe_2_ and InSe devices. On the other hand, a robust p-n junction based on BP-PdSe_2_ heterojunctions has been reported [74]. In this device, asymmetric contact engineering was employed, where the BP side was deposited with Cr/Au contacts and the PdSe_2_ side was deposited with Sc/Au contacts. This approach provided a robust rectification behavior, with further tunability achieved by varying the back-gate voltage. The BP-PdSe_2_ heterojunction device exhibited a broadband response from 532 nm to 1310 nm, with a high responsivity (>10^5^ A/W) and external quantum efficiency (>10^6^%). The device also demonstrated a high detectivity reaching 10^13^ Jones, indicating its potential for highly sensitive photodetection applications.

#### 4.2.3. Graphene-Sandwiched PdSe_2_-2D Heterostructure-Based Photodetectors

While the use of lithographically patterned contacts has been the norm for lateral heterojunction-based photodetectors, utilizing van der Waals contacts like graphene for vertical heterojunction-based photodetectors can be an efficient approach. The shorter channel length in vertical devices, on the order of nanoscales, enables faster charge carrier extraction and separation, increasing the device response speed and responsivity.

**Figure 18:**
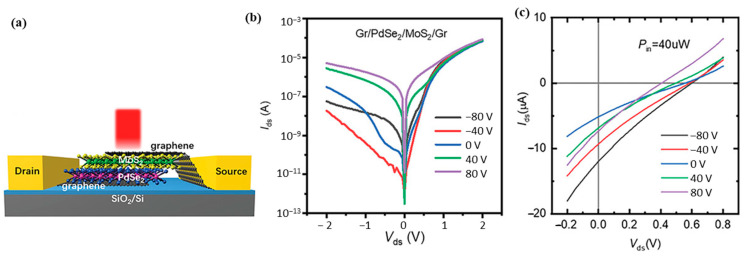
(**a**) Schematic of the graphene-sandwiched PdSe_2_/MoS_2_ vdWH photodetector. (**b**) Output curves at various gate voltages from −80 to 80 V. (**c**) Photoresponse characteristics of the device at various gate voltages with notable *V_oc_* and *I_sc_*. Adapted with permission from ref. [75] © 2023 Wiley-VCH GmbH.

Zhong et al. [52] reported the fabrication of self-powered, graphene-sandwiched PdSe_2_-MoSe_2_ vertical photodetectors. These devices exhibited a rectification ratio greater than 10^3^ under dark conditions, and fast response times of 41.7 μs (rise) and 62.5 μs (fall) due to the short transit channel of approximately 57 nm for the photogenerated carriers. The devices demonstrated a remarkable responsivity of 651 mA/W under self-bias conditions, with a broad spectral response covering the visible to near-infrared (NIR) regime. Furthermore, the responsivity could be enhanced to 1.16 A/W under a reverse bias of −1 V, and the rectification ratio could be tuned by adjusting the back-gate voltage, which modifies the Schottky barrier height. Building on a similar strategy, Chen et al. [75] fabricated graphene-sandwiched PdSe_2_-MoS_2_ and PdSe_2_-WS_2_ vertical heterojunction photodetectors. These devices exhibited a large open-circuit voltage of 0.6 V under 650 nm illumination. Both devices showed enhanced performance, including higher rectification ratios and faster response times, by tuning the back-gate voltage. (Figure 18a–c).

These studies highlight the advantages of using van der Waals contacts, such as graphene, in vertical heterojunction-based photodetectors. The short carrier transit channels and efficient charge separation facilitated by the vertical device architecture enable the realization of high-performance, broadband photodetectors with fast response times and tunable characteristics.

A summary of some of the best-performing PdSe_2_-based photodetectors and their key performance parameters has been presented in Table 2, as found from the literature.

## 5. Outlook and Perspective

Van der Waals Two-dimensional (2D) palladium diselenide (PdSe_2_) exhibits unique properties that present significant opportunities for future optoelectronic applications. The layer-dependent modulation of its bandgap, coupled with high ambipolar mobility and pronounced anisotropic broadband light absorption, positions PdSe_2_ as an ideal candidate for next-generation transistor and photodetector technologies. Furthermore, its exceptional thermal stability at room temperature enhances its suitability for thermoelectric and bolometric applications, thereby facilitating ultra-broadband sensing capabilities. The latest advancements in photodetectors based on PdSe_2_ have showcased its significant promise for optoelectronic applications. PdSe_2_ possesses distinctive characteristics, including a wide range of spectral sensitivity up to THz, exceptional responsivity, and rapid response times, making it a highly promising 2DLM for the forthcoming era of high-performance photodetectors.

The versatility of the two-step selenization method for large-area PdSe_2_ film growth, coupled with the ability to integrate PdSe_2_ into hybrid photodetector structures, has enabled the development of high-performance, multifunctional photodetector devices with tailored spectral and polarization-sensitive responses. Additionally, the implementation of standard metal deposition techniques allow fabrication of an array of photodetectors, showing potential for future commercial applications. Research is still open on this stem of the tree as reported cases for the large area PdSe_2_ are polycrystalline and the highly crystalline phase is the desired industry need for commercial application of large area in-plane integrated optoelectronic devices. With continued research effort in this stem, acceptable device-to-device/area-area variance along with stability and good repeatability will make PdSe_2_ a strong candidate for back-end-of-line (BEOL) applications, like all other 2DLM.

Prospects of PdSe_2_ nanosheets onto SOI ridge waveguides provide a promising platform for the development of high-performance, integrated photodetectors operating in the important telecommunications wavelength range. The combination of high responsivity and large bandwidth makes these devices attractive for a variety of applications in communication and signal processing systems. The incorporation of mechanically exfoliated PdSe_2_ into heterojunction device topologies, such as PdSe_2_-InSe and PdSe_2_-(TMDC) structures, have resulted in improved performance of photodetectors. These heterostructures have demonstrated the ability to detect a wide range of wavelengths, can be controlled by gate voltage, exhibit outstanding performance metrics, including high responsivity and detectivity, and can be suitable candidates for future miniaturized spectrometers. Noteworthy, the exceptionally high ambipolar mobility of PdSe_2_ as a channel material plays a crucial role in determining the ultrafast response speed in these lateral heterojunction-based photodetectors. Furthermore, the utilization of van der Waals contacted/graphene-sandwiched, vertical PdSe_2_-based heterojunctions has displayed significant potential as well. The compact nanoscale carrier transit channels present in these vertical devices facilitate effective charge separation and rapid response times, appealing for photodetection applications that need high speed and high sensitivity.

Continued research on PdSe_2_-based photodetector is expected to result in significant performance enhancements through the optimization of device design, material engineering, and integration strategies. The key focus area of research should include in-depth investigations in the field of metal contact-2DLM interface as high contact resistance is still the major bottleneck for 2DLM-based photodetectors and their inferior mobility and charge carrier extraction efficiency. The adaptability of PdSe_2_ and its ability to work well with different heterostructure setups indicate that these photodetectors will have a significant impact on upcoming optoelectronic technologies, including imaging, sensing, and communication systems.

## Figures and Tables

**Table 1:** Figure of merits (FoMs) for characterizing PdSe_2_-based photodetectors.

FoM Parameter	Definition	Expression	Unit
**Responsivity**	Ratio of Photocurrent (*I_photo_*) to the incident light power (*P_in_*).	$R=\frac{I_{photo}}{P_{in}}=\frac{I_{light}-I_{dark}}{P_{in}}$	A/W
**External Quantum Efficiency**	Ratio of number of photogenerated electrons per unit time to the number of incident photons. Also referred to as the product of charge transfer efficiency and light absorption efficiency. [*h* is the Planck constant, *e* is the electronic charge, *c* is the light speed, and *λ* is the incident wavelength of light source]	$EQE=\frac{N_{I}}{N_{P}}=\frac{h\;\cdot c\cdot I_{photo}}{e\cdot \lambda \cdot P_{in}}$	-
**Dark Current**	Current persistent in the photodetector under dark/no light conditions.	$I_{dark}$	A
**Gain**	Ratio of number of photogenerated e-h pairs collected by the contacts to the number of photoexcited e-h pairs. [*µ* is the carrier mobility, *V_bias_* is the applied bias, *L* is the channel length, *τ_life_* is the carrier lifetime, and *τ_transit_* is the carrier transit time.]	$G=\frac{\tau_{life}}{\tau_{transit}}=\frac{µ\cdot \tau_{life}}{L^{2}}V_{bias}$ $\left[\tau_{transit}=\frac{L^{2}}{{µ\cdot V}_{bias}}\right]$	-
**Linear Dynamic Range**	It is the range of illumination log power density for which the log range of photocurrent shows linearity, before reaching saturation.	$LDR=20log\frac{P_{sat}}{P_{low}}$	dB
**Response time**	The rise (decay) time is defined as the time for the photocurrent to reach 10–90% (90–10%), after introduction (removal) of incident photon flux.	$\tau_{rise}(\tau_{decay})$	s
**3dB bandwidth @** **Relative balance**	The cut-off frequency *f_3dB_*, of modulated incident light when the responsivity of the photodetector decreases by *3dB* (0.707 of the stable value). At a much lower frequency, the responsivity is independent of the light modulation frequency. [*I_max_* and *I_min_* are the maximum and minimum photocurrent]	$f_{3dB}@\frac{I_{max}-I_{min}}{I_{max}}\%$	Hz
**Noise Current**		$i_{N}$	AHz^−1/2^
**Noise Equivalent Power**	The minimum light power that is detectable by a detector is defined as the light power when the signal-to-noise ratio (SNR) is 1. [*A* is the effective device area.]	$NEP=\frac{i_{N}}{R}=\frac{\sqrt{A}}{D^{*}}$	WHz^−1/2^
**Measured Specific Detectivity**	It is a measure of a detector’s ability to resolve the weakest of the incident light signal	$D^{*}=\frac{\sqrt{A}}{NEP}=\frac{\sqrt{A}}{i_{N}}R$	cmHz^1/2^W^−1^ (Jones)
**Calculated Specific Detectivity**	Specific detectivity is calculated, assuming that the major contribution of noise is from dark current shot noise.	$D^{*}=\sqrt{\frac{A}{2\cdot q\;\cdot I_{dark}}}R$	cmHz^1/2^W^−1^ (Jones)

**Table 2:** Key performance metrics influencing the performance of PdSe_2_-based photodetectors.

Channel Material [Fabrication Method] {Structure}	Bandwidth [Operation]	Mobility (cm^2^V^−1^s^−1^) Electron {Hole}	Contact Types	On/Off Ratio	Response Time (ms) Rise/Fall [Wavelength]	Responsivity (AW^−1^) [Wavelength]	Detectivity (Jones) [Wavelength]	Ref
PdSe_2_ [M.E.-Flake] {Waveguide}	1260 nm–1565 nm [P.C.]	216	Ti/Au (B.C.)	-	-	1.758 [1550 nm]	-	[71]
PdSe_2_ [M.E.-Flake] {FET}	405 nm–1.249 mm [P.C./P.V.]	121	Ni/Au (T.C.) Cr/Au (T.C.)	-	7.5 × 10^−3^	5 × 10^−3^ [1.249 mm]	1.84 × 10^12^ [1850 nm]	[22]
PdSe_2_ [M.E.-Flake] {FET}	532 nm–4.05 µm [P.C./P.G.]	4	Cr/Au (T.C.)	-	-	708 [1064 nm]	1.31 × 10^9^ [1064 nm]	[70]
PdSe_2_ [M.E.-Flake] {FET}	1064 nm [P.T.E.]	92	Ti/Au (T.C.)	10^4^	0.156/0.163	-	-	[76]
PdSe_2_ [M.E.-Flake] {FET}	9.3 µm [P.B.]	-	Cr/Au (T.C.)	-	210/230	245 × 10^−3^ [9.3 µm]	-	[77]
PdSe_2_-MoS_2_ [M.E.-Flake] {L.H. FET}	450 nm–10.6 µm [P.C./P.G.]	138.9 {57.7}	Pd/Au (T.C.)	10^3^	74.5/91.3 [10.6 µm]	42.1 [10.6 µm]	1.10 × 10^9^ [10.6 µm]	[72]
PdSe_2_-InSe [M.E.-Flake] {L.H. FET}	532 nm–1650 nm [P.C.]	-	Ti/Au (T.C.)	3.8 × 10^4^	53/72 [532 nm]	161 [1250 nm]	1 × 10^10^ [1650 nm]	[73]
BP-PdSe_2_ [M.E.-Flake] {L.H. FET}	532 nm–1310 nm [P.V./P.G.]	-	Cr/Au and Sc/Au (T.C.)	9.5 × 10^5^	1.6/4.7 [1310 nm]	9.6 × 10^5^ [532 nm]	5.8 × 10^13^ [532 nm]	[74]
Gr-PdSe_2_-MoS_2_-Gr [M.E.-Flake] {V.H.}	650 nm [P.V.]	-	Au-vdW (T.C.)	10^7^	0.129_/_0.146	0.173 [650 nm]	6.7 × 10^11^ [650 nm]	[75]
Gr-PdSe_2_-MoSe_2_-Gr [M.E.-Flake] {V.H.}	405 nm–1060 nm [P.V.]	-	Cr/Au (B.C.)	5.6 × 10^3^	41.7 × 10^−3^/ 62.5 × 10^−3^	0.651 [532 nm]	5.29 × 10^11^ [532 nm]	[52]
PdSe_2_ [CVD-Flake] {FET}	405 nm–940 nm [P.T.E.]	-	Au-vdW (T.C.)	2.8 × 10^2^	4 × 10^−3^/ 14 × 10^−3^	1.4 × 10^−3^	2.55 × 10^7^	[20]
PdSe_2_ [CVD-Flake] {FET}	365 nm–2200 nm [P.B.]	-	In/Au (T.C.)	-	1000/1200	37.6 × 10^−3^ [1550nm]	-	[65]
PdSe_2_-MoS_2_ [CVD-M.E. Flake] {V.H.}	405 nm–1000 nm [P.C.]	-	Au (T.C.)	10^2^	378/708 [830 nm]	6.9 [830 nm]	6.3 × 10^10^ [830 nm]	[67]
PdSe_2_-WS_2_ [CVD] {V.H. FET}	532 nm–1550 nm [P.C./P.G.]	3.5 × 10^−2^ {2.7 × 10^−4^}	Ti/Au (B.C.)	-	49/90 [635 nm]	0.019 × 10^−3^	-	[68]
BPQD-PdSe_2_-Si [CVD-Spin coating] {0D-2D-3D Hybrid V.H.}	200 nm–3044 nm [P.V.]	-	Au (T.C.) In-Ga (B.C.)	10^5^	38 × 10^−3^/ 44 × 10^−3^	300.2 × 10^−3^	1.18 × 10^13^	[49]
PdSe_2_-GaN [CVD] {2D-3D Hybrid L.H.}	200 nm–370 nm [P.V.]	-	Au and Ni/Au (T.C.)	1.4 × 10^3^	28.5 × 10^−3^/ 122.6 × 10^−3^	249.9 × 10^−3^ [360 nm]	7.9 × 10^12^	[66]
PdSe_2_-Cs-doped FAPbI_3_ [CVD-Spin coating] {2D-Perovskite Hybrid L.H.}	200 nm–1550 nm [P.V.]	{4.75}	Au (T.C.)	10^4^	3.5 × 10^−3^/ 4 × 10^−3^	313 × 10^−3^	10^13^	[54]
PdSe_2_-SiNW [CVD] {2D-3D Hybrid V.H.}	200 nm–4.6 µm [P.V.]	-	Au (T.C.) In-Ga (B.C.)	10^2^	25.1 × 10^−3^/ 34 × 10^−3^	726 × 10^−3^	3.19 × 10^14^	[51]
PdSe_2_-Si Pyramid [CVD] {2D-3D Hybrid V.H.}	980 nm–1650 nm [P.V.]	-	Au (T.C.) In-Ga (B.C.)	1.6 × 10^5^	N.A.	456 × 10^−3^ [980 nm]	9.97 × 10^13^ [980 nm]	[78]
PdSe_2_-GeNCs CVD 2D-3D Hybrid V.H.	980 nm–1650 nm [P.V.]	-	Gr-Ag (T.C.) In-Ga (B.C.)	5	25.4 × 10^−3^/ 38.5 × 10^−3^ [1550 nm]	530.2 × 10^−3^ [1550 nm]	1.45 × 10^11^ [1550 nm]	[79]

M.E.—Mechanical Exfoliation, L.H.—Lateral Heterostructure, V.H.—Vertical Heterostructure, T.C.—Top Contact, B.C.—Bottom Contact, P.C.—Photoconductive, P.V.—Photovoltaic, P.G.—Photogating, P.B.—Photobolometric, P.T.E.—Photothermoelectric.

## Data Availability

Not applicable.
